# Trends in Lower-Risk Gambling by Age and Net Income among Finnish Men and Women in 2011, 2015, and 2019

**DOI:** 10.1007/s10899-024-10355-x

**Published:** 2024-10-01

**Authors:** Tanja Grönroos, Jukka Kontto, Matthew M. Young, David C. Hodgins, Anne H. Salonen

**Affiliations:** 1 Finnish Institute for Health and Welfare, Department of Public Health and Welfare, P.O. Box 30, Helsinki, FI-00271 Finland; 2 Faculty of Social Sciences, University of Helsinki, P.O. Box 18, Helsinki, FI-00014 Finland; 3 Greo Evidence Insights, Guelph, ON N1H 7T8 Canada; 4 Canadian Centre on Substance Use and Addiction, Ottawa, ON K1P 5E7 Canada; 5 Department of Psychology, Carleton University, Ottawa, ON K1S 5B6 Canada; 6 Department of Psychology, University of Calgary, Calgary, AB T2N 1N4 Canada; 7 Faculty of Health Sciences, University of Eastern Finland, Kuopio, Finland

**Keywords:** Gambling, Population survey, Gender, Socio-economic factors, Lower-risk gambling guidelines

## Abstract

Lower-risk Gambling Guidelines (LRGGs) were developed in Canada to reduce the risk of gambling-related harm. The LRGGs, published in 2021, consist of three limits: gamble no more than 1% of household income per month; gamble no more than four days per month; and avoid regularly gambling at more than two types of games. All three limits should be followed at the same time. This study focuses on the situation in Finland before the LRGGs were published. The aim of this study is to investigate trends in lower-risk gambling by age and net income among men and women in the Finnish adult population in 2011, 2015, and 2019. Data were drawn from cross-sectional Finnish Gambling population surveys, including permanent residents in Mainland Finland aged 15–74 with Finnish, Swedish or Sámi as their mother tongue (2011; *n* = 4,484, 2015; *n* = 4,515, and 2019; *n* = 3,994). The results showed an increase in the prevalence of lower-risk gambling, rising from 29% in 2011 to 39% in 2019. This upward trend was observed among both men and women, with the prevalence among men increasing from 23 to 33%, and among women from 34 to 45%. The lowest prevalence of lower-risk gambling was found among individuals aged 60–74, especially regarding expenditure guidelines, as well as among women in the lowest income tertile. In conclusion, although the prevalence of lower-risk gambling has increased in Finland, there is still potential for further improvement to minimize harm.

**Supplementary Information:**

The online version contains supplementary material available at 10.1007/s10899-024-10355-x.

## Introduction

Gambling is a growing public health issue with diverse, harmful impacts at the individual, family, community, and society level (Latvala et al., 2019; Wardle et al., 2019). According to the Conceptual Framework of Harmful Gambling (Abbott et al., 2018), any type of gambling can potentially lead to negative outcomes. The harms can be short- or long-term and their intensity can vary from minor to significant. The factors related to the risks and effects of harmful gambling are multifaceted, ranging from the general (cultural, social, psychological, and biological) to the specific (gambling environment, gambling exposure, gambling types, and gambling resources). Although the greatest amount of personal gambling-related harm is suffered by those with problem gambling (incl. gambling disorder), most of the total gambling-related harm is distributed among people gambling who do not meet the definition of a “problem gambler” (Browne et al., 2018; Browne et al., 2020; Volberg et al., 2021). From the public health perspective, societal effects of gambling are therefore largely attributable to the harms experienced by people gambling at “at-risk” levels. It is noteworthy that the efforts targeted at the prevention of harmful gambling, rather than problem gambling, may potentially be more effective as they can impact a larger group of people (Currie et al., 2006, 2009).

### The Lower-Risk Gambling Guidelines

To fill the lack of evidence-based guidelines about how to gamble in a way that poses minimal risk, the Lower-Risk Gambling Guidelines (LRGGs) were developed by the Canadian Centre on Substance Use and Addiction (CCSA) (Young et al., 2021, 2022; Hodgins et al., 2023; Canadian Centre on Substance Use and Addiction, 2024). These guidelines, which were published in 2021, are a set of quantitative limits, and information about special risk populations, contextual factors and other health messages that should be followed in order to gamble in a lower-risk manner. The LRGGs consist of three limits: gamble no more than 1% of household income per month; gamble no more than four days per month; and avoid regularly gambling at more than two types of games. All three limits should be followed at the same time to minimize the risk of experiencing gambling-related harm.

Development of the LRGGs spanned 4 years and included analysis of data from over 60,000 people who gamble from eight different countries, including Finland. Because the project funding and core research team were based in Canada, much of the other development work was conducted in Canada. This included consultation with a pan-Canadian multi-sectorial advisory committee, input from a national online survey (*n* = 10,000), and interviews with people who gamble. Additional work included a systematic literature review and meta-analysis assessing special risk populations and contextual factors associated gambling harm (Allami et al., 2021) and a literature review assessing the influence of alcohol and other substances on gambling behaviour. This work resulted in six scientific publications (Hodgins et al., 2023; Young et al., 2022; Currie et al., 2019; Allami et al., 2021; Currie et al., 2020; Flores-Pajot et al., 2021) and a suite of knowledge mobilization (KmB) products such as downloadable banner ads, posters, and an online self-assessment tool permitting people to assess the risk level of their own gambling (Canadian Centre on Substance Use and Addiction, 2024).

The quantitative recommended limits on expenditure, frequency, and gambling types have been found to be consistent with previous research and can predict future harm from gambling (Dowling et al., 2021). They are useful for public health promotion and can also be used to monitor risk of harm at the population level. Currently, research is underway to test the feasibility of using the LRGGs in the Finnish cultural context to prevent and reduce the gambling-related harm among the population.

### Socio-Demographic and Socio-Economic Differences in Gambling Harms

Within the general population, some groups experience more harmful gambling than others. Men and young adults are often found to be the most vulnerable (William et al., 2012; Ekholm et al., 2014). There is also evidence middle-aged women experience harmful gambling, especially among those who play electronic gaming machines (EGMs) (Hing et al., 2016), although, a recently published meta-analysis did not support this (Allami et al., 2021). Compared to men, women tend to start gambling later in their life and progress more quickly from the gambling onset to experiencing gambling problems. This reflects to a telescoping progress (Syvertsen et al., 2023). In Finland, the prevalence of problem gambling (SOGS 3+) among women has increased in the last decade (Salonen et al., 2020).

Evidence also suggests that gambling harms are disproportionately experienced by socio-economically disadvantaged groups (Sharman et al., 2019). Low education is found to be associated with harmful gambling (Ekholm et al., 2014). Furthermore, harmful gambling is more common among those who have received social security benefits. This is especially the case among those who have received income support, which is a last resort form of financial aid in Finland. (Latvala et al., 2021.) In addition, harmful gambling is more prevalent among unemployed (Latvala et al., 2021). Higher income is found to be associated with higher total gambling expenditure (Grönroos et al., 2022). However, lower-income individuals spend a higher proportion of their income on gambling and are also at higher risk of experiencing harms (Castrén et al.,2018; Roukka & Salonen, 2020). Overall, those with gambling problems often face other challenges as well, such as health problems and other addictions (Lorains et al.,^2011^; Dowling et al., 2015; Håkansson et al., 2018; Grönroos et al., 2024). These problems can be further exacerbated by gambling.

In this paper, the focus is on the situation in Finland before the LRGGs were published in 2021. This study describes the proportion of individuals gambling above and below each of the quantitative limits included in the guidelines as well as the proportion of gambling above and below all three limits together. Previous literature suggests that gambling participation and harms among men and women may differ in terms of age and socio-economic factors. In this study, separate analyses are therefore conducted for men and women, as combined analysis may obscure gender distinctions due to the stronger effect sizes of men. The aim of this study is to investigate trends in lower-risk gambling by age and net income among men and women in the Finnish adult population in 2011, 2015, and 2019. The overall prevalence of gambling in the population was stable over this period.

## Methods

### Design, Participants, and Data Collection

The cross-sectional data were extracted from the Finnish Gambling population studies, which were conducted by the Finnish Institute for Health and Welfare (Salonen et al., 2020). The surveys assessed gambling, problem gambling, and attitudes and opinions towards gambling. The participants were randomly selected from the National Population Information System covering the total population in Finland. Based on that, invitation letters were sent to 16,000 persons in 2011, 7,400 in 2015, and 7,800 in 2019. The inclusion criteria were: (1) aged 15 to 74; (2) Finnish, Swedish or Sámi as first language; and (3) resident in Mainland Finland.

In 2011, the study was described to the potential participants as a ‘gambling and health survey’ and data were collected by Taloustutkimus, a private market research company. In 2015 and 2019, the study was described to the potential participants as a survey on ‘gambling and opinions on gambling’, with the data collected by Statistics Finland. The data (*n* = 12,993) were collected as computer-assisted telephone interviews. Ultimately, responses were obtained from 4,484 participants in 2011, 4,515 participants in 2015, and 3,994 participants in 2019. The response rates were 28%, 62%, and 52%, respectively. For this present study, only those who have a legal right to gamble (i.e., 18–74-year-olds) in Finland were included. The data represents the entire Finnish population.

### Measures

#### Prevalence of Gambling no More than 1% of Individual Net Income

Gambling expenditure was measured in 2011 and 2015 as follows: ‘Roughly how much money do you spend on gambling in a typical week (€)?’. In 2019, gambling expenditure was inquired with the question: ‘Think about the past 12 months. Estimate the amount of money that you spent on gambling on average per week, per month or during the year (€).’ All gambling expenditure figures were transformed into monthly gambling expenditure. In the LRGGs (Young et al., 2021; Hodgins et al., 2023), household net income is the first limit (gamble no more than 1% of household net income per month). However, individual net income was used as household net income was not available. Furthermore, individual net income is better suited to the Nordic context, as Finnish couples typically maintain their own financial accounts rather than share them with their spouses (Pepin & Cohen, 2021). Then, based on monthly gambling expenditure and individual monthly net income, “gamble no more than 1% of individual net income per month” (yes/no) was created.

#### Prevalence of Gambling No More Than Four Days per Month

Gambling frequency was asked for 18 game types. The list included games provided by the Finnish gambling monopoly company Veikkaus Oy. In addition, offshore games and games offered in Åland and in ferries between Finland, Sweden, and Estonia were included in the list. Gambling days per month were calculated as follows. First, gambling frequency for each game type was transformed into days: 1) daily or almost daily = 30/month; several times a week = 16/month; once a week = 4/month; 2 to 3 times a month = 2.5/month; once a month = 1/month; and less than monthly = 0.25/month. In 2019, gambling frequency was measured by four categories, instead of six. Therefore, in 2019, gambling frequency for each game type was transformed into days: 1) daily or several times a week = 19/month; once a week = 4/month; 1 to 3 times a month = 2/month; and less than monthly = 0.25/month. Then, the dichotomic “gamble no more than four days per month” (yes/no) was calculated based on the overall gambling days per month.

#### Prevalence of Gambling No More Than Two Game Types per Month

The dichotomic “Avoid regularly (monthly) gambling at more than two types of games” (yes/no) was created based on the gambling frequency of 18 game types.

#### Prevalence of Lower-Risk Gambling

Prevalence of lower-risk gambling within the Finnish population was assessed by calculating how many people gambled below all three limits included in the LRGGs.

#### Socio-demographic and Socio-economic Factors

Socio-demographic and socio-economic factors included gender, age, and individual net income tertiles.

### Statistical Analysis

The data were weighted based on gender, age, and region of residence and weighted prevalence of gambling measures were calculated for each year. The T-test was used for calculating the confidence intervals for prevalences and to evaluate whether the prevalences of 2019 were different from the prevalences of other years. Logistic regression models were constructed to examine whether age or income modified the association between year and gambling measure. Separate analyses were conducted for men and women by age and income tertiles. Respondents under 18-years of age were excluded from the analysis. Statistical analysis was conducted using IBM SPSS Statistics software version 27.0 (IBM Corp. Released, 2020) and R version 4.3.2 (R Core Team, 2023).

## Results

Prevalence of lower-risk gambling among all Finns changed across the years, being 28.7% in 2011, 29.7% in 2015, and 39.3% in 2019 (Table 1). Among men, the prevalence of lower-risk gambling was 23.1%, 27.5%, and 33.3%, respectively. Among women, the corresponding figures were 34.4%, 31.8%, and 45.4%, respectively. The limit-specific results showed that the prevalence of lower-risk gambling was higher in 2019 than in previous years. The prevalence of gambling no more than four days per month did not change statistically significantly between 2011 and 2019 among women, and between 2015 and 2019 among men.

**Table 1:** Prevalence of Finnish men and women gambling below the lower-risk gambling limits in 2011, 2015, and 2019 (%)

	2011 (*N* = 4,484)		2015 (*N* = 4,515)		2019 (*N* = 3,994)		T-test *p*-value 2011 vs. 2019	T-test *p*-value 2015 vs. 2019
	%	N	%	N	%	N		
**All**								
max 1% of individual net income	58.5	2,058	58.2	1,697	67.6	1,920	<0.001	<0.001
max 4 days per month	60.5	2,082	60.6	2,159	63.3	1,953	0.021	0.022
max 2 types of games	86.9	3,955	87.2	3,941	91.0	3,628	<0.001	<0.001
below all the limits	28.7	1,256	29.7	1,322	39.3	1,534	<0.001	<0.001
**Men**								
max 1% of individual net income	47.1	806	51.4	839	59.7	912	<0.001	<0.001
max 4 days per month	50.3	865	52.2	1,016	54.1	895	0.025	0.262
max 2 types of games	80.0	1,729	80.5	1,868	86.6	1,758	<0.001	<0.001
below all the limits	23.1	472	27.5	625	33.3	670	<0.001	<0.001
**Women**								
max 1% of individual net income	70.5	1,252	66.6	858	76.5	1,008	<0.001	<0.001
max 4 days per month	72.2	1,217	69.9	1,143	73.4	1,058	0.446	0.032
max 2 types of games	93.8	2,226	93.9	2,073	95.4	1,870	0.023	0.025
below all the limits	34.4	784	31.8	697	45.4	864	<0.001	<0.001

The percentages were calculated from the weighted data. Statistical significance (p) calculated using the T-test

### Prevalence of Lower-Risk Gambling by Age

**Fig. 1:**
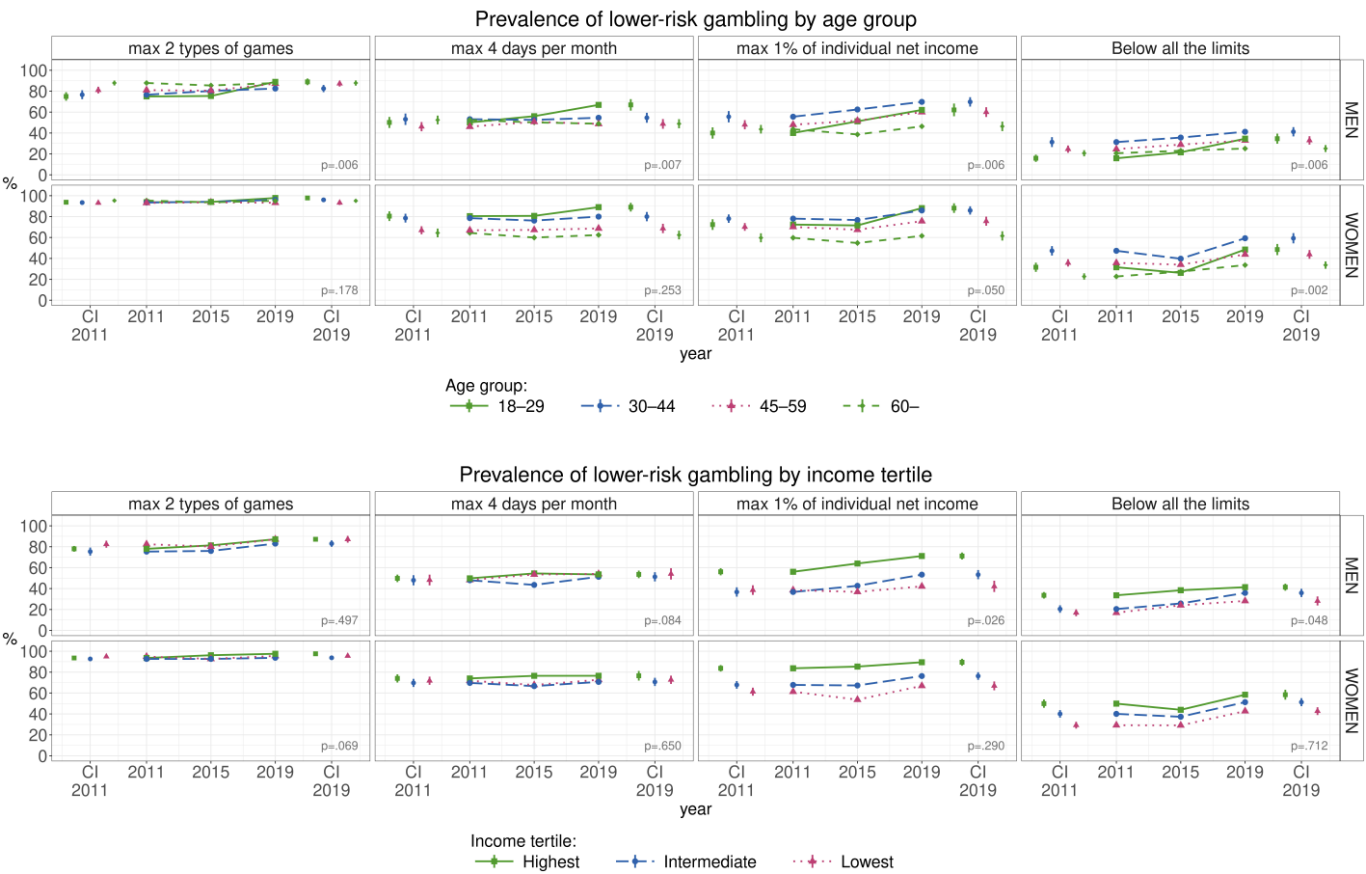
Prevalence of Finnish men and women gambling below the lower-risk limits by age and individual income between 2011 and 2019 (%). The percentages were calculated from the weighted data. CI = Confidence Interval

In 2019, the prevalence of lower-risk gambling among men was of lowest among 60–74-years-olds (25.1%; 95% CI 21.8–28.5) (Fig. 1; Supplementary File 1). The prevalence of lower-risk gambling among men increased especially among 18–29-years-olds, being 15.9% (95% CI 12.4–19.3) in 2011 and 34.6% (95% CI 29.7–39.4) in 2019. Apart from men aged 60–74, the prevalence of lower-risk gambling increased among all age groups between 2011 and 2019.

In 2019, prevalence of lower-risk gambling among women was lowest among 60–74-years-olds (33.6%; 95% CI 30.1–37.1) and highest among 30–44-years-olds (59.3%; 95% CI 54.4–64.2) (Fig. 1; Supplementary File 1). Similar to men, among women the prevalence of lower-risk gambling increased especially among 18–29-years-olds, being 31.5% (95% CI 27.1–35.9) in 2011 and 48.5% (95% CI 43.1–53.8) in 2019. Additionally, in all other age groups, the prevalence of lower-risk gambling increased between 2011 and 2019.

### Prevalence of Lower-Risk Gambling by Income

In 2019, the prevalence of lower-risk gambling among men was lower among the lowest income tertile (28.2%; 95% CI 23.8–32.5) than in the highest income tertile (41.3%; 95% CI 38.0–44.7) (Fig. 1; Supplementary File 2). The prevalence of lower-risk gambling among men, however, did not differ statistically significantly between the lowest and intermediate income tertile. The prevalence of men gambling below all the three LRGGs increased across all income tertiles between 2011 and 2019.

In 2019, the prevalence of lower-risk gambling among women was lowest among the lowest income tertile (42.7%; 95% CI 39.0–46.4) (Fig. 1; Supplementary File 2). The prevalence of women gambling below all the three LRGGs increased in the lowest and intermediate income tertiles, but there was no statistically significant change in the highest income tertile.

## Discussion

This paper describes the prevalence of lower-risk gambling among the Finnish population by age and net income in 2011, 2015, and 2019. The limits and the trends in them were observed each on its own and together, as recommended by the CCSA (Young et al., 2021). Overall, the prevalence of lower-risk gambling among all Finns was 39.3% in 2019. Further, a positive change was observed since the prevalence of lower-risk gambling has increased from 2011 (28.7%) and from 2015 (29.7%). This is positive as such an increase indicates that, over the assessed time period, a greater proportion of the Finnish population gambled at levels of gambling involvement that were at low risk of gambling related harm. This finding is in line with the previous Finnish Gambling study results based on the South Oaks Gambling Screen (SOGS; Salonen et al., 2020). While at-risk gambling (SOGS = 1–2) decreased from 13.3 to 10.7%, the prevalence of problem gambling (SOGS = 3+) remained unchanged at around 3% during this time. It is noteworthy, that at the same time past-year gambling prevalence has been rather stable in Finland, being 77.9% in 2011, 80.0% in 2015 and 78.4% in 2019 (Salonen et al., 2020).

### Lower-Risk Gambling by Gender

As expected, the prevalence of lower-risk gambling was higher among women than among men. Several previous studies have shown that men gamble more often and have more gambling problems than women (Ekholm et al., 2012; Dowling et al., 2017; Salonen et al., 2022). Men also tend to spend more money on gambling than women (Grönroos et al., 2022). Furthermore, men also have different game type preferences compared with women. In Finland men gamble more on all types of games compared with women, except for the scratch cards (Salonen et al., 2020). Finnish men also gamble offshore more often than Finnish women (Hagfors et al., 2023), and offshore online gambling has increased between 2007 and 2019 (Salonen et al., 2022). Despite this, the prevalence of lower-risk gambling increased between 2011 and 2019 among men. Among women, however, the prevalence of lower-risk gambling decreased between 2011 and 2015, while the prevalence of lower-risk gambling increased from 2015 to 2019 considerably.

### Lower-Risk Gambling by Age and Income

Another finding of this study was that the prevalence of lower-risk gambling has clearly increased among 18–29-year-olds. The result was in line with the Finnish Gambling study results indicating a reduction in at-risk gambling (SOGS = 1–2) among 19–24-years-olds (Salonen et al., 2020). At the same time, attitudes towards gambling changed more negative particularly among 18–34-year-old men. This trend may have been partly influenced by the fact that the age limit of gambling in Finland was raised from 15 to 18 in October 2010. Therefore, many in this age group have grown up in different type of gambling environment than previous generations. EGMs were given a slightly longer transition time and the new age limit for EGMs took effect on July 2011. At the time, the aim was also to improve the supervision of age limits and prohibit gambling advertisements directed to under 18-years-olds. Interestingly, the prevalence of lower-risk gambling was shown not to be of lowest among the youngest age group. This finding is somewhat surprising given that at-risk gambling is highest among 18–34-years-olds (Salonen et al., 2020). It should be noted that the type of gambling in which a person regularly engages is a strong predictor of gambling problems. The risk is found to be highest in online gambling and gambling EGMs or poker (Allami et al., 2021). This type of gambling is rather common among young age groups; however, all types of underaged gambling has decreased since 2011, except for gambling scratch cards (Salonen et al., 2020).

The prevalence of lower-risk gambling was lowest among 60–74-years-olds, particularly for the limit related to gambling expenditure. In this age group, most common game types include weekly lottery games (70%), scratch cards (39%), slot machines outside the casino (18%) and daily lottery games (24%), while other game types are gambled only rarely (Salonen et al., 2020). Furthermore, 60–74 year-olds can be quite diverse in their backgrounds; some are still at work, and some are already pensioners. However, it can be assumed that a large number are already retired because in Finland a common retirement age is around 63 years. A declined income in retirement may partly explain the lower proportion of lower-risk gambling among the oldest age group, in particular in the guideline related to gambling expenditure relative to income. In retirement, some individuals may continue their previous level of gambling despite a reduction in income (Landreat et al., 2019). Further, it is noteworthy that the negative consequences of gambling may be particularly significant for older adults. As already mentioned, many older people have a lower income due to retirement, and thus have fewer financial resources to recover from social, financial, and, especially, health-related consequences resulting from problematic gambling (Levens et al., 2005; Landreat et al., 2019).

Among women, the prevalence of lower-risk gambling was of lowest in the lowest income tertile in 2019. This was not a surprising result as gambling problems are found to be more common among those with lower income (Latvala et al., 2021). In addition, although those with high income spend more on gambling, those with lower income contribute proportionally more (Beckert & Lutter, 2009; Canale et al., 2016; Castrén et al., 2018; Roukka & Salonen, 2020). EGMs have been identified as a single most harmful game type (Binde et al., 2017; Russell et al., 2023). In fact, it has also been proven in Finland that that the density of EGMs is higher in areas with a lower socio-economic level than in areas with a higher socio-economic level (Raisamo et al., 2019).

### Finnish Gambling Policy / Environment

In addition to the increase in the age limit for gambling, the Finnish gambling environment has changed in other ways over the last decade as a result of other policy changes as well as transnational gambling trends. Formerly, the Finnish three-party monopoly system changed in early 2017, since the three operators were merged into a single company, Veikkaus Oy. At the time, Veikkaus Oy opened a new online gambling site with an easy access to a large number of different gambling activities. On the other hand, mandatory identification, which allowed the use of several gambling management tools, were introduced first online and later on land-based. Firstly, in 2017, new restrictions and gambling management tools (including game loss limits, money transfer limits, and the possibility of a game ban) were provided on Veikkaus Oy’s new online gambling site. These online gambling restriction tools, some of which are mandatory and some of which are voluntary, may have particularly affected the amount of money spent on gambling, which may be seen in the increase in the prevalence of lower-risk gambling, especially in the prevalence of spending no more than 1% of individual income.

The percentage of Finns reporting they had gambled online gambling during the last decade increased, with the highest growth between 2015 and 2019 (Salonen et al., 2020). As shown in several studies, online gamblers are more likely to report harmful gambling than land-based gamblers (Griffiths et al., 2009; Wood & Williams, 2011; Canale et al., 2016; Pallesen et al., 2021; Allami et al., 2021). Nevertheless, lower-risk gambling has simultaneously increased. A recently published Finnish study (Lind et al., 2022) showed that online gambling was not linked to higher problem gambling prevalence. This may be partly due to the Finnish gambling context which is characterized by a high prevalence of land-based EGMs in everyday locations such as grocery stores and petrol stations. Therefore, the particularly harmful nature of land-based gambling in Finland may partly overshadow the negative consequences of online gambling (Lind et al., 2022). Thus, the increase in online gambling may not have affected lower-risk gambling among the general population as much as in other countries.

Moreover, despite of increased gambling availability due to online gambling, gambling has become more occasional in Finland; gambling less than monthly has increased, whereas gambling at least once a month has decreased (Salonen et al., 2020). This change may be due to adaption, which is related to gambling exposure, one of the gambling-specific factors depicted in the Conceptual Framework of Harmful Gambling (Abbott et al., 2018). In the framework, adaption means that, over time, populations adapt to gambling exposure and people participate less in gambling and experience less gambling-related harm, even though gambling exposure continues to increase. In Finland, those who gamble may have adapted to the large number of different gambling activities at least at some extent and, thus, increased gambling availability may not have affected to gambling involvement – thus the observed increase in prevalence of lower-risk gambling.

In addition, it is noteworthy that LRGGs do not take into account the number of player accounts in different online gambling sites. In other words, it is possible for a person to gamble only one or two game types, but on several different gambling sites, leading possibly to greater gambling. Consideration of this issue is timely, as the government of Finland reforms the Finnish gambling system by opening it up to competition through a licensing system by first of January 2026. In principle, online casino games and online betting would be covered by the licencing system. In 2019, those Finns who had gambled non-monopoly games, had an average of 2.7 different player accounts. Around 30% of them had three or more accounts. The large number of player accounts is found to be linked to gambling problems. (Salonen et al., 2020).

Several other potential factors may have influenced the increase in the prevalence of lower-risk gambling among Finns. During recent years, the public debate on gambling and Veikkaus Oy has been exceptionally active and critical, which may have affected Finns’ gambling and attitudes towards gambling (Macey et al., 2023). Also, long-term work on preventing and reducing gambling-related harm in Finland may have had an effect.

### Strengths and Limitations

There are some limitations in this study, which should be taken into consideration when interpreting the results. This study utilized self-reported data, as in most gambling studies (Shaffer et al., 2010). A few studies have evaluated the accuracy of self-reported data with actual data provided by gambling operator (Braverman et al., 2014; Auer & Griffiths, 2017). These studies showed that respondents tend to underestimate the amount of money they spent on gambling (Braverman et al., 2014; Auer & Griffiths, 2017). However, it should be noted that self-reported loss is still found to correlate with the actual loss (Auer & Griffiths, 2017). In addition, the respondents are not found to consistently indicate a favourable distortion of their gambling losses or gains, as they underestimated or overestimated their gambling outcomes (Braverman et al., 2014).

In order to ensure cross-sectional comparability, an effort was made to ensure the design and the questionnaire remained as similar as possible throughout the trend series. However, in 2011, the data were collected by Taloustutkimus, and in 2015 and 2019 the survey was conducted in collaboration with Statistics Finland. Moreover, the data for 2011 and 2019 were collected in autumn/winter, while the data for 2015 was collected in the spring. Moreover, the response rate varied throughout the years, which may affect comparisons of data collected in different years. In 2019, the respondents reported their gambling expenditure based on gambling frequency of their choice. In earlier years, the questions concerning gambling expenditure were worded slightly differently. Despite some differences, the combined data forms coherent data that enables trend research, covering important information on Finnish gambling for a decade.

This study did not examine the impact of game form on whether a person gambled according to the LRGGs. However, previous studies have shown that some forms of gambling (e.g., electronic gaming machines, poker, casino games) are more closely associated with problem gambling than other forms (e.g., lotteries) (Binde et al., 2017). Fast-paced games that involve frequent betting can more quickly lead to problems than discontinuous forms of gambling. With these fast-paced games such as electronic gaming machines, people can spend large amounts of money in a short time. In future studies, it should be important to investigate how participation with different types of games affect the prevalence of lower-risk gambling.

Lastly, this study evaluated gambling from the public health perspective and the results were encouraging. On the other hand, it is noteworthy that this type of evaluation does not give an insight from the perspective of those suffering from the most severe gambling harms. Above mentioned changes, in the gambling environment such as 24/7 availability, increase in online and particularly offshore gambling, may have impacted differently those gambling extensively.

## Conclusions

Before the LRGG were published and implemented, the prevalence of lower-risk gambling among all Finns was 39.3% in 2019. Although the overall past-year gambling prevalence remained unchanged between 2011 and 2019, the prevalence of lower-risk gambling increased during this period. This suggests that a greater proportion of people than before are gambling in ways that reduce the risk of harm from gambling. Despite this, there is still room for improvement and interventions targeting at-risk gambling. Implementation of the LRGG might further enhance this positive trend. A group of multi-professional Finnish experts translated and prepared the LRGG materials for the Finnish cultural context in 2023. Currently, the feasibility of the LRGGs is being tested in order to clarify the LRGG’s potential for implementation in Finland. Our results imply that, if/when the LRGG will be implemented in Finland, 60–74-years-olds and persons with low income might benefit the most out of it.

## Electronic Supplementary Material

Below is the link to the electronic supplementary material.

Supplementary Material 1

Supplementary Material 2
