# Supplementary material for: Trends in Lower-Risk Gambling by Age and Net Income among Finnish Men and Women in 2011, 2015, and 2019
Source: J Gambl Stud. 2024 Oct 1;41(1):267–81. doi: 10.1007/s10899-024-10355-x (PMC11861171; doi:10.1007/s10899-024-10355-x)
Supplement: Supplementary file 1 — Supplementary Material 1 [file 10899_2024_10355_MOESM1_ESM.docx]

Table 1 Prevalence of Finnish men and women gambling below the lower-risk limits by age between 2011 and 2019 (%)

|  | 2011  (N = 4,484) | | | 2015  (N = 4,515) | | | 2019  (N = 3,994) | | |
| --- | --- | --- | --- | --- | --- | --- | --- | --- | --- |
|  | % | N | 95% CI | % | N | 95% CI | % | N | 95% CI |
| Men |  |  |  |  |  |  |  |  |  |
| max 1% of individual net income |  |  |  |  |  |  |  |  |  |
| 18–29 | 40.0 | 320 | 34.6–45.4 | 51.2 | 257 | 45.0–57.3 | 62.1 | 249 | 56.0–68.2 |
| 30–44 | 55.6 | 328 | 50.2–61.0 | 62.5 | 437 | 57.9–67.1 | 69.8 | 406 | 65.3–74.3 |
| 45–59 | 47.9 | 508 | 43.6–52.3 | 51.8 | 488 | 47.4–56.3 | 60.0 | 427 | 55.3–64.7 |
| 60–74 | 43.6 | 557 | 39.5–47.8 | 38.6 | 483 | 34.2–43.0 | 46.4 | 467 | 41.8–50.9 |
| max 4 days per month |  |  |  |  |  |  |  |  |  |
| 18–29 | 50.1 | 349 | 44.8–55.4 | 56.1 | 358 | 50.9–61.3 | 66.9 | 287 | 61.4–72.4 |
| 30–44 | 53.2 | 324 | 47.7–58.6 | 52.6 | 484 | 48.1–57.0 | 54.6 | 421 | 49.8–59.4 |
| 45–59 | 46.1 | 496 | 41.7–50.5 | 50.7 | 567 | 46.5–54.8 | 48.7 | 453 | 44.1–53.3 |
| 60–74 | 52.5 | 556 | 48.3–56.6 | 50.1 | 541 | 45.9–54.4 | 48.9 | 513 | 44.6–53.2 |
| max 2 types of games |  |  |  |  |  |  |  |  |  |
| 18–29 | 75.0 | 434 | 70.9–79.1 | 75.4 | 440 | 71.4–79.5 | 88.9 | 377 | 85.8–92.1 |
| 30–44 | 76.6 | 377 | 72.3–80.9 | 80.2 | 543 | 76.9–83.6 | 82.5 | 477 | 79.1–85.9 |
| 45–59 | 81.0 | 590 | 77.9–84.2 | 80.5 | 647 | 77.4–83.5 | 87.2 | 536 | 84.4–90.1 |
| 60–74 | 87.8 | 716 | 85.4–90.2 | 85.4 | 675 | 82.8–88.1 | 87.8 | 640 | 85.3–90.3 |
| below all the limits |  |  |  |  |  |  |  |  |  |
| 18–29 | 15.9 | 434 | 12.4–19.3 | 21.5 | 440 | 17.7–25.4 | 34.6 | 377 | 29.7–39.4 |
| 30–44 | 31.3 | 377 | 26.6–36.0 | 35.6 | 543 | 31.6–39.7 | 41.2 | 477 | 36.8–45.6 |
| 45–59 | 24.6 | 590 | 21.1–28.1 | 29.0 | 647 | 25.5–32.5 | 32.9 | 536 | 29.0–36.9 |
| 60–74 | 20.6 | 716 | 17.7–23.6 | 23.1 | 675 | 19.9–26.2 | 25.1 | 640 | 21.8–28.5 |
|  |  |  |  |  |  |  |  |  |  |
| Women |  |  |  |  |  |  |  |  |  |
| max 1% of individual net income |  |  |  |  |  |  |  |  |  |
| 18–29 | 72.4 | 302 | 67.3–77.4 | 71.5 | 168 | 64.7–78.4 | 88.1 | 196 | 83.5–92.7 |
| 30–44 | 78.0 | 409 | 74.0–82.1 | 76.7 | 303 | 71.9–81.5 | 85.9 | 303 | 81.9–89.8 |
| 45–59 | 70.1 | 592 | 66.4–73.8 | 67.4 | 372 | 62.6–72.2 | 75.6 | 382 | 71.3–79.9 |
| 60–74 | 59.7 | 507 | 55.4–64.0 | 54.7 | 474 | 50.3–59.2 | 61.5 | 467 | 57.1–66.0 |
| max 4 days per month |  |  |  |  |  |  |  |  |  |
| 18–29 | 80.4 | 283 | 75.8–85.1 | 80.7 | 255 | 75.8–85.5 | 89.0 | 220 | 84.8–93.2 |
| 30–44 | 78.5 | 366 | 74.3–82.8 | 76.2 | 382 | 71.9–80.4 | 80.0 | 315 | 75.5–84.4 |
| 45–59 | 66.9 | 570 | 63.1–70.8 | 67.3 | 457 | 63.0–71.6 | 68.6 | 415 | 64.2–73.1 |
| 60–74 | 64.4 | 503 | 60.2–68.6 | 59.9 | 572 | 55.9–63.9 | 62.4 | 522 | 58.2–66.5 |
| max 2 types of games |  |  |  |  |  |  |  |  |  |
| 18–29 | 93.7 | 431 | 91.4–96.0 | 94.0 | 401 | 91.7–96.3 | 97.8 | 339 | 96.2–99.4 |
| 30–44 | 93.4 | 476 | 91.2–95.6 | 94.2 | 487 | 92.1–96.2 | 96.0 | 394 | 94.1–97.9 |
| 45–59 | 93.1 | 719 | 91.2–94.9 | 93.6 | 590 | 91.6–95.6 | 93.2 | 540 | 91.1–95.3 |
| 60–74 | 95.3 | 741 | 93.8–96.8 | 93.6 | 732 | 91.9–95.4 | 95.1 | 691 | 93.5–96.7 |
| below all the limits |  |  |  |  |  |  |  |  |  |
| 18–29 | 31.5 | 431 | 27.1–35.9 | 26.2 | 401 | 21.8–30.5 | 48.5 | 339 | 43.1–53.8 |
| 30–44 | 47.3 | 476 | 42.8–51.8 | 39.7 | 487 | 35.3–44.1 | 59.3 | 394 | 54.4–64.2 |
| 45–59 | 35.7 | 719 | 32.2–39.2 | 34.0 | 590 | 30.2–37.9 | 43.8 | 540 | 39.6–48.0 |
| 60–74 | 22.7 | 741 | 19.7–25.7 | 27.3 | 732 | 24.1–30.6 | 33.6 | 691 | 30.1–37.1 |

CI = Confidence Interval. The percentages were calculated from the weighted data.
