# Supplementary material for: Trends in Lower-Risk Gambling by Age and Net Income among Finnish Men and Women in 2011, 2015, and 2019
Source: J Gambl Stud. 2024 Oct 1;41(1):267–81. doi: 10.1007/s10899-024-10355-x (PMC11861171; doi:10.1007/s10899-024-10355-x)
Supplement: Supplementary file 2 — Supplementary Material 2 [file 10899_2024_10355_MOESM2_ESM.docx]

Table 2 Prevalence of Finnish men and women gambling below the lower-risk limits by individual income between 2011 and 2019 (%)

|  | 2011  (N = 4,484) | | | 2015  (N = 4,515) | | | 2019  (N = 3,994) | | |
| --- | --- | --- | --- | --- | --- | --- | --- | --- | --- |
|  | % | N | 95% CI | % | N | 95% CI | % | N | 95% CI |
| Men |  |  |  |  |  |  |  |  |  |
| max 1% of individual net income |  |  |  |  |  |  |  |  |  |
| Lowest | 38.5 | 434 | 34.0–43.1 | 36.8 | 358 | 31.8–41.8 | 42.1 | 328 | 36.7–47.5 |
| Intermediate | 36.7 | 449 | 32.3–41.2 | 42.6 | 523 | 38.4–46.9 | 53.3 | 497 | 48.9–57.7 |
| Highest | 56.1 | 830 | 52.7–59.4 | 63.9 | 784 | 60.6–67.3 | 71.1 | 717 | 67.8–74.5 |
| max 4 days per month |  |  |  |  |  |  |  |  |  |
| Lowest | 48.2 | 370 | 43.1–53.3 | 53.5 | 388 | 48.5–58.5 | 54.0 | 330 | 48.6–59.4 |
| Intermediate | 47.9 | 409 | 43.1–52.8 | 43.5 | 545 | 39.3–47.7 | 51.2 | 499 | 46.8–55.6 |
| Highest | 49.8 | 752 | 46.2–53.3 | 54.5 | 832 | 51.1–57.9 | 53.5 | 722 | 49.9–57.2 |
| max 2 types of games |  |  |  |  |  |  |  |  |  |
| Lowest | 82.4 | 492 | 79.1–85.8 | 80.1 | 474 | 76.5–83.7 | 87.1 | 423 | 83.9–90.3 |
| Intermediate | 75.4 | 485 | 71.5–79.2 | 76.0 | 615 | 72.6–79.4 | 83.0 | 568 | 79.9–86.1 |
| Highest | 78.0 | 888 | 75.3–80.7 | 81.3 | 941 | 78.8–83.8 | 87.2 | 846 | 84.9–89.4 |
| below all the limits |  |  |  |  |  |  |  |  |  |
| Lowest | 16.9 | 492 | 13.5–20.2 | 24.0 | 474 | 20.2–27.9 | 28.2 | 423 | 23.8–32.5 |
| Intermediate | 20.4 | 485 | 16.8–24.0 | 25.8 | 615 | 22.3–29.2 | 35.8 | 568 | 31.9–39.8 |
| Highest | 33.6 | 888 | 30.4–36.7 | 38.4 | 941 | 35.3–41.5 | 41.3 | 846 | 38.0–44.7 |
|  |  |  |  |  |  |  |  |  |  |
| Women |  |  |  |  |  |  |  |  |  |
| max 1% of individual net income |  |  |  |  |  |  |  |  |  |
| Lowest | 61.3 | 620 | 57.4–65.1 | 53.6 | 470 | 49.1–58.2 | 66.9 | 486 | 62.7–71.1 |
| Intermediate | 67.8 | 629 | 64.1–71.4 | 67.3 | 557 | 63.3–71.2 | 76.3 | 520 | 72.6–79.9 |
| Highest | 83.7 | 561 | 80.6–86.7 | 85.3 | 290 | 81.2–89.4 | 89.4 | 329 | 86.1–92.8 |
| max 4 days per month |  |  |  |  |  |  |  |  |  |
| Lowest | 71.8 | 515 | 67.9–75.7 | 67.7 | 550 | 63.8–71.6 | 72.7 | 492 | 68.7–76.6 |
| Intermediate | 69.7 | 546 | 65.8–73.6 | 66.7 | 641 | 63.0–70.3 | 70.6 | 522 | 66.7–74.6 |
| Highest | 74.0 | 482 | 70.0–77.9 | 76.5 | 349 | 72.0–80.9 | 76.5 | 330 | 71.9–81.1 |
| max 2 types of games |  |  |  |  |  |  |  |  |  |
| Lowest | 94.9 | 757 | 93.3–96.5 | 92.7 | 731 | 90.8–94.5 | 95.5 | 678 | 93.9–97.1 |
| Intermediate | 92.6 | 709 | 90.7–94.6 | 92.6 | 787 | 90.8–94.4 | 93.7 | 659 | 91.9–95.6 |
| Highest | 93.5 | 621 | 91.6–95.5 | 96.2 | 451 | 94.4–98.0 | 97.6 | 421 | 96.1–99.0 |
| below all the limits |  |  |  |  |  |  |  |  |  |
| Lowest | 29.2 | 757 | 26.0–32.5 | 29.1 | 731 | 25.8–32.4 | 42.7 | 678 | 39.0–46.4 |
| Intermediate | 40.1 | 709 | 36.4–43.7 | 37.3 | 787 | 33.9–40.7 | 51.3 | 659 | 47.5–55.1 |
| Highest | 49.9 | 621 | 45.9–53.8 | 44.0 | 451 | 39.4–48.6 | 58.4 | 421 | 53.7–63.2 |

CI = Confidence Interval. The percentages were calculated from the weighted data.
